# Correction: Technical traditions and individual variability in the Early Neolithic: Linear pottery culture flint knappers in the Aisne Valley (France)

**DOI:** 10.1371/journal.pone.0292774

**Published:** 2023-10-05

**Authors:** The PLOS ONE Staff

There are errors in the Funding section. The correct Funding statement is: This research was carried out in the framework of the following projects: (i) ANR Homes, project n° ANR-18-CE27-0011, Caroline Hamon dir., CNRS, UMR 8215 and (ii) the Operational Programme Research, Development, and Education–Project “Postdoc2MUNI” (No. CZ.02.2.69/0.0/0.0/18_053/0016952). The funders had no role in study design, data collection and analysis, decision to publish, or preparation of the manuscript.

The publisher apologises for the error.
