# Supplementary material for: Technical traditions and individual variability in the Early Neolithic: Linear pottery culture flint knappers in the Aisne Valley (France)
Source: PLoS One. 2022 Jun 1;17(6):e0268442. doi: 10.1371/journal.pone.0268442 (PMC9159609; doi:10.1371/journal.pone.0268442)
Supplement: S1 File — (PDF) [file pone.0268442.s001.pdf]

site maison	NB	fosse	MP	inventR pièce	tailleurs	longueur
BLH_120	1	124	SEN	2727	2'	45,94
BLH_120	1	122	SEN	68	3	38,61
BLH_120	1	124	SEN	191	3	41,72
BLH_120	1	124	TERT	1064	3	23,13
BLH_120	1	122	TUR	1102	3	37,65
BLH_120	1	124	TERT	1494	3	35,05
BLH_120	1	124	SEN	144	3	35,93
BLH_120	1	124	TERT	926	3	68,5
BLH_120	1	124	QUA	821	1'	31
BLH_120	1	122	QUA	177	1'	30
BLH_120	1	124	TERT	1609	1'	39,53
BLH_120	1	124	TERT	1519	1'	18,26
BLH_120	1	124	SEN	595	1'	23,9
BLH_120	1	124	SEN	1010	1'	39,97
BLH_120	1	124	SEN	636	1'	38,03
BLH_120	1	122	SEN	528	1'	31,29
BLH_120	1	122	SEN	81	1'	51,24
BLH_120	1	124	SEN	592	1'	38,13
BLH_120	1	124	TERT	599	1	26,31
BLH_120	1	122	SEN	70	1	77,09
BLH_120	1	124	GRIS	2727	1''	53,64
BLH_120	1	124	SEN	823	1''	62,87
BLH_120	1	122	TUR	1724	1'	23,99
CCF_380	1	382	QUA	45231	2	44,24
CCF_380	1	382	TERT	45491	2	26,04
CCF_380	1	382	TERT	45511	2	67,67
CCF_380	1	378	TERT	48305	2	67,04
CCF_380	1	378	TERT	44342	2	58,42
CCF_380	1	382	TERT	45282	2	41,55
CCF_380	1	-	SEN	48307	2	37,13
CCF_380	1	382	TERT	45365	2	29,46
CCF_380	1	382	SEN	45228	2	46,44
CCF_380	1	378	TERT	43XX6	2	33,38
CCF_380	1	378	TERT	43471	2	42,6
CCF_380	1	378	TUR	48303	2	48,48
CCF_380	1	378	SEN	6622		
CCF_380	1	382	TERT	45536	2	41,28
CCF_380	1	378	TERT	44560	2	32,18
CCF_380	1	382	SEN	46038	2	40,88
CCF_380	1	382	SEN	45230	1'	41,3
CCF_380	1	382	TERT	45261	2	58,79
CCF_380	1	382	TERT	45446	2	44,68
CCF_380	1	378	SEN	48302	INDET	34,41
CCF_380	1	378	TERT	43138	2	40,56
CCF_380	1	382	TERT	45366	2	41,6
CCF_380	1	378	TERT	44343	2	43
CCF_380	1	378	TERT	48297	2	46,92
CCF_380	1	378	SEN	44763	1'	79,49
CCF_380	1	378	TERT	44603	2	34,79
CCF_380	1	378	TERT	44463	1	28,91

CCF_380	1	378	TERT	44240	2	44,84
CCF_380	1	382	TERT	45422	1'	43,25
CCF_380	1	378	SEN	43127	1	42,31
CCF_380	1	378	TERT	43142	1	35,64
CCF_380	1	378	TERT	7017	1	31,38
CCF_380	1	378	TERT	43185	2	38,48
CCF_380	1	378	TUR	43163	1'	46,99
CCF_380	1	378	TERT	6242	1	49,18
CCF_380	1	378	TERT		1'	37,25
CCF_380	1	378	TERT	6240	1	45,23
CCF_380	1	377 ?	TERT	6812	2	67,51
CCF_380	1	378	TERT	44659	1	41,09
CCF_380	1	378	TERT	6722	1	48,25
CCF_380	1	378	SEN	43132	1	71,36
CCF_380	1	378	SEN	7018		
CCF_380	1	378	SEN	48303	2	24,86
CCF_380	1	382	SEC	45260	1	36,61
CCF_380	1	378	TERT	43492	INDET	32,1
CCF_380	1	378	TERT	44080	1	30,53
CCF_380	1	378	TERT	43489	1	36,43
CCF_570	1	556	SEN	49457	2'	32,69
CCF_570	1	598	SEN	49503	2'	33,01
CCF_570	1	556	SEN	49385	2'	31,22
CCF_570	1	556	SEN	49346	3	56,45
CCF_570	1	556	SEN	49478	2'	29,73
CCF_570	1	556	SEN	49453	2'	30,47
CCF_570	1	556	SEN	49451	4	58,67
CCF_570	1	556	TERT	49724	2'	90,65
CCF_570	1	556	SEN	49312	2'	34,53
CCF_570	1	556	TERT	49480	2'	39,54
CCF_570	1	556	SEN	49375	3	31,42
CCF_570	1	556	SEN	49356	3	41,98
CCF_570	1	556	TERT	49336	3	35,77
CCF_570	1	598	SEN	49498	3	35,05
CCF_570	1	556	SEN	49383	3	64,43
CCF_570	1	556	SEN	49372	1'	43,32
CCF_570	1	556	SEN	4937449378	3	44,9
CCF_570	1	556	SEN	49340	3	33,23
CCF_570	1	556	SEN	49419	2'	37,46
CCF_570	1	556	SEN	49445	3	28,61
CCF_570	1	556	SEN	49398	3	30,29
CCF_570	1	556	TERT	49311	3	31,97
CCF_570	1	556	TERT	49328	3	38,92
CCF_570	1	598	SEN	49496	4	33,24
CCF_570	1	556	SEN	49344	3	67,12
CCF_570	1	598	TERT	49495	3	17,74
CCF_570	1	556	SEN	49308		
CCF_570	1	556	SEN	49363	4	39,75
CCF_570	1	556	SEN	49405	1	77,01
CCF_570	1	556	TERT	49467	3	72,15

largeur	épaisseur	fragmentat°	type support	section	régularité	corniche
19,1	3,34	mp	Lp	3	1	1
16,78	4,02	p	Lp	3	1	2
22,88	4,75	p	Lp	3	1	2
18,66	5,06	p	Lp	3	1	2
16,2	4,03	p	Lp	2	1	2
18,9	4,66	p	Lp	4	1	2
13,82	3,64	p	Lp	3	1	2
15,59	6,09	mp	Lp	3	3	2
22	4	p	Lp	2	1	3
23	4	p	Lp	3	1	3
12,73	3,26	p	Lp	3	3	3
13,11	2,31	p	Lp	3	1	3
12,04	3,15	p	Lp	3	1	3
12,52	3,82	p	Lp	3	3	3
18,4	2,92	p	Lp	3	1	3
21,74	5,53	p	Lp	2	1	3
16,98	2,53	p	Lp	3	2	3
17,62	2,96	p	Lp	3	2	3
12,66	3,3	p	Lp	3	1	3
18,02	5,11	e	Lp	2	3	3
17,69	4,62	p	Lp	3	0	3
11,86	4,4	e	Lp	2	0	3
16,42	5,12	p	Lp	3	1	3
24,2	6,11	p	LpL4	3	2	1
24,44	7,12	p	Lp	3	1	1
29,19	5,78	p	Lp	3	1	NAT
14,97	4,38	p	Lp	3	2	NAT
23,69	5,49	p	Lp	2	2	NAT
17,57	5,67	p	L2	2	NR	NAT
19,35	4,18	p	L4	2	1	1
26,44	6,6	p	Lp	3	1	1
24	5,85	p	Lp	3	1	1
20,82	6,32	p	Lp	2	1	1
22,39	4,81	p	Lp	2	1	1
20,38	5,95	p	Lp	3	2	1
		p	L4	2	1	1
19,31	8,79	p	L1	2	2	1
18,22	3,94	p	Lp	3	NR	1
18,08	5,42	p	Lp	2	1	2
22,63	9,55	p	Lp	2	3	2
25,2	7,2	p	Lp	2	1	2
16,1	6,19	p	L2	2	1	2
18,96	5,11	p	Lp	3	1	2
23,73	4,47	p	Lp	3	1	2
22,57	7,64	p	Lp	3	1	2
23,82	5,5	p	Lp	2	1	2
36,47	9,27	p	Lp	3	2	2
16,12	3,77	e	Lp	3	1	2
20,84	6,06	p	Lp	3	1	2
18,26	4,31	p	Lp	2	1	2

18,43	3,59	p	Lp	3	1	2
14,2	6,94	p	Lp	3	1	3
19,9	5	p	L2	3	1	3
25,85	6,11	p	Lp	2	1	3
14,8	4,85	p	Lp	3	1	3
25,93	5,21	p	Lp	3	1	3
23,17	9,53	p	L4	3	3	3
19,56	5,57	p	Lp	3	1	3
13,53	6,42	p	Lp	3	1	3
15,9	5,2	p	Lp	3	1	3
13,85	5,42	e	Lp	2	1	3
17,9	5,69	p	L4	3	1	3
19,77	3,33	p	Lp	3	1	3
15,22	8,03	e	Lp	2	2	3
		p	Lp	3	2	3
15,42	4,42	p	Lp	3	1	3
22,6	7,16	p	Lp	3	1	3
30,19	5,49	p	L4	3	1	3
20,64	4,46	p	Lp	2	1	3
26,69	8,94	p	Lp	3	1	3
27,22	4,19	p	Lp	3	1	NAT
22,44	5,97	p	L2	2	1	NAT
16,2	5,04	p	Lp	3	1	NAT
14,61	6,05	p	L4	2	3	NAT
20,15	4,65	p	L4	3	2	NAT
12,52	3,2	p	Lp	3	1	NAT
14,7	6,76	e	L2	2	2	1
11,68	6,27	e	Lp	4	2	1
13,2	5,12	p	L4	2	1	1
18,07	6,21	p	L4L2	2	2	1
23,12	4,83	p	Lp	3	1	2
21,11	6,24	p	Lp	2	1	2
12,97	3,8	p	Lp	3	1	2
15,18	4,68	p	Lp	3	1	2
12,7	5,04	e	Lp	3	1	2
18,56	5,33	p	Lp	3	1	3
19,01	4,66	p	Lp	2	1	3
21,75	6,59	p	Lp	3	1	3
13,52	4,6	p	L1	2	2	3
22,41	4,12	p	Lp	3	1	3
21,3	5	p	L2	2	1	3
18,31	4,96	p	Lp	2	1	3
17,78	5,34	p	L2	3	1	3
23,04	4,51	p	L4	2	2	3
20,69	4,41	e	Lp	3	3	3
22,11	5,24	p	Lp	2	1	3
		p	Lp	3	1	3
14,51	4,36	p	Lp	3	2	3
21,04	4,36	e	L4	-	2	3
18,28	7,14	e	Lp	3	1	3

talon	demi-lune	larg.talon	ép.talon	angle chasse	code	outils
1	1	10,38	5,06	90	321	1
1	0	10,77	3,51	90	212	1
1	0	10,32	4,35	90	212	1
1	0	8,27	1,96	90	212	
1	0	5,97	2,71	90		1
3	0	11,74	2,55	80	1234	
1	0	6,46	3,27	90	212	1
1	1	9,43	4,17	70	123	
1	0	8,81	2,01	70°		
1	0	5,26	1,75	70°	321	
7	0	2,16	0,79	NR	212	
6	0	5,03	1,37	80	212	
6	0	5,58	1,2	70	123	
1	0	8,68	1,6	70	123	1
6	0	6,49	0,99	70	123	
6	0	6,02	1,51	70		
7	0	1,91	1,31	70	212	
6	0	5,62	1,14	NR		
6	0	5,04	1,01	70	212	
1	0	5,66	1,71	90		
4	0	5,7	1,83	90	212	1
4	0	6,93	1,98	90		1
RET	0	NR	2,98	90	212	1
1	0	8,12	4,77	90	321	1
1	1	11,04	4,09	90	212	1
1	0	8,36	3,73	90	2123	1
1	0	9,33	5,6	90	321	1
2	0	12,23	4,43	90		
3	0	9,24	4,6	90		
1	1	10,36	5,66	80		1
1	1	13,63	7,07	90	321	1
1	0	9,83	4,04	70	212	1
1	0	7,98	3,75	70		1
1	0	10	3,16	90		
3	0	24,41	11,53	95	212	1
1	1	10	6	80		1
1	1	10,55	7,4	90		
1	0	8,29	5,08	80		
1	0	5,54	1,75	90		1
2	0	5,95	3,2	80		1
1	0	15,8	4,57	90		
1	1	10,6	5,3	80		1
2	0	7,96	3,46	90	212	1
3	0	16,98	5,07	90	123	1
2	0	9,81	3,26	80	212	1
1	0	11,91	5,52	90		
1	0	15,04	6,96	90	212	1
1	0	6,25	1,61	90	212	
3	0	10,46	4,73	90	321	
1	1	8,64	3,05	90		

1	0	8,4	3,2	90	123	
1	0	6,56	2,4	70	212	
1	0	7,06	2,94	70	212	1
6	0	6,1	2,07	90		1
2	0	4,71	2,62	80	212	1
1	0	18,47	4,48	80	212	1
1	0	7,64	2,53	95	123	1
7	0	3,14	1,78	90	123	1
1	0	8,01	3,65	70	321	1
7	0	4,19	2,1	90	212	1
2	0	8,56	4,47	90		1
1	0	5,89	2,34	90	212	
1	0	7,34	2,12	90	212	
1	0	4,8	1,98	80		
1	0	6	2	90	212	1
1	0	7,75	2,97	90	212	
3	0	7,26	2,53	90	212	
1	0	14,38	5,29	80	321	
1	0	11,89	3,92	80		
1	0	7,74	1,48	80	212	
2	0	11,26	3,22	80	321	1
1	0	5,24	2,4	80		1
1	0	7,44	3,4	90	212	1
1	0	5,88	1,4	90		1
1	0	7,21	2,41	90	321	1
1	0	4,66	2,08	90	212	1
1	0	4,72	2,1	90		
1	0	5,76	2,53	90	2123	
1	0	4,94	1,93	90		1
2	0	7,08	4,11	90		
1	0	7,3	2,08	90	212	
2	0	6,66	2,51	90		1
1	1	6,8	3,69	80	321	
3	0	10,15	4,35	90	212	1
2	0	5,26	2,39	80	212	1
3	0	7,08	2,68	90	212	
2	0	6,41	2,85	95		
3	0	9,94	4,34	90	212	
1	1	11,59	7,01	90		1
1	0	4,81	1,67	80	212	1
1	0	5,28	2,7	90		1
1	1	6,35	2,05	90		1
1	0	5,64	2,15	90	321	1
2	0	9,75	4,51	80		
7	0	3,5	1,8	90	123	
1	0	4,65	2,36	90		1
2	0	5	4	80	212	1
2	0	5,6	3,57	90	123	1
3	0	7,44	2,8	80		
3	0	7,25	2,57	80	212	1
